# Targeting the MEK1/2 pathway to combat *Staphylococcus aureus* infection and inflammation in cystic fibrosis

**DOI:** 10.1128/mbio.00775-25

**Published:** 2025-05-27

**Authors:** Eryn Zuiker, Gregory Serpa, Mithu De, Yiwei Liu, Daniel J. Wozniak, Kymberly M. Gowdy, Jean Charron, Susan E. Birket, Megan R. Kiedrowski, Emily A. Hemann, Matthew E. Long

**Affiliations:** 1 Division of Pulmonary, Critical Care, and Sleep Medicine, Department of Internal Medicine, College of Medicine, The Ohio State University Wexner Medical Center, Columbus, Ohio, USA; 2 Department of Microbial Infection and Immunity, College of Medicine, The Ohio State University Wexner Medical Center, Columbus, Ohio, USA; 3 Dorothy M. Davis Heart and Lung Research Institute, The Ohio State University Wexner Medical Center 271931, Columbus, Ohio, USA; 4 Department of Microbiology, The Ohio State University 215854 , Columbus, Ohio, USA; 5 Centre de Recherche du Centre Hospitalier Universitaire de Québec-Université Laval (Oncology Axis), Quebec City, Quebec, Canada; 6 Département de Biologie Moléculaire, Biochimie Médicale and Pathologie, Université Laval, Quebec City, Quebec, Canada; 7 Division of Pulmonary, Allergy, and Critical Care, Department of Medicine, Heersink School of Medicine, The University of Alabama at Birmingham 654677, Birmingham, Alabama, USA; 8 Gregory Fleming James Cystic Fibrosis Research Center, University of Alabama at Birmingham, Birmingham, Alabama, USA; The University of Mississippi Medical Center, Jackson, Mississippi, USA; The Children's Hospital of Philadelphia, Philadelphia, Pennsylvania, USA

**Keywords:** *Staphylococcus aureus*, cystic fibrosis, macrophages, MEK1/2

## Abstract

*Staphylococcus aureus* infections remain an ongoing challenge for people with cystic fibrosis (PwCF), with the increased global prevalence of multidrug-resistant strains requiring new therapeutic approaches. Our previous studies demonstrated anti-inflammatory effects of several MEK1/2 inhibitor compounds, including PD0325901, CI-1040, and trametinib, in human phagocytes from PwCF and a murine *S. aureus* pulmonary infection model (M. De, G. Serpa, E. Zuiker, K. B. Hisert, et al., Front Cell Infect Microbiol 14:1275940, 2024, https://doi.org/10.3389/fcimb.2024.1275940). A recently developed MEK1/2 inhibitor compound, ATR-002, has been recognized for its ability to exert direct antibacterial effects on gram-positive bacterial species, including *S. aureus* (C. Bruchhagen, M. Jarick, C. Mewis, T. Hertlein, et al., Sci Rep 8:9114, 2018, https://doi.org/10.1038/s41598-018-27445-7). However, whether ATR-002 elicits antibacterial effects on clinically relevant strains of *S. aureus* or anti-inflammatory effects is unknown. In this study, the effects of ATR-002 on human CF macrophage TLR2-induced pro-inflammatory cytokine secretion were evaluated, demonstrating that ATR-002 reduced TNF-α and IL-8 secretion induced by the TLR2 agonists FSL-1 or Pam3CSK4. The antibacterial effects of ATR-002 were evaluated by minimum inhibitory concentration testing using *S. aureus* clinical isolates obtained from PwCF. Utilization of a murine methicillin-resistant *S. aureus* (MRSA) pulmonary infection model further confirmed the *in vivo* anti-inflammatory and antibacterial effects of ATR-002. Finally, infection of wild-type and *Mek2^KO^* mice revealed that loss of MEK2 was host-protective during MRSA pulmonary infection by reducing neutrophil-mediated inflammation without altering bacterial clearance. In summary, this study highlights the therapeutic potential of targeting the MEK1/2 pathway to combat MRSA pulmonary infections.

**IMPORTANCE:**

*Staphylococcus aureus* infections pose a significant burden on global healthcare systems. Community-associated transmission of methicillin-resistant *S. aureus* (MRSA) and the increasing prevalence of other drug-resistant *S. aureus* isolates limit therapeutic options to combat this opportunistic pathogen. Infection-induced inflammation is a significant driver of tissue damage, especially in cystic fibrosis pulmonary infections. However, therapeutic strategies that can reduce inflammation without compromising host defense and bacterial clearance mechanisms are lacking. This study investigates the dual anti-inflammatory and antibacterial effects of a MEK1/2 inhibitor as a therapeutic strategy to target both host and pathogen with a single compound. This work also identifies host MEK2 as a specific target that can be modulated to reduce inflammation without impairing host defense against MRSA pulmonary infection. Results from this study can inform future human clinical trials to evaluate the ability of the MEK1/2 inhibitor compound ATR-002 to both combat *S. aureus* infections and reduce inflammation that accompanies these infections.

## INTRODUCTION

Cystic fibrosis (CF) is a genetic disease caused by mutations in the cystic fibrosis transmembrane conductance regulator (CFTR). Impaired CFTR function establishes a pulmonary niche that favors chronic and recurrent pulmonary bacterial infections, resulting in excessive inflammation and tissue damage. The CF pulmonary microenvironment is especially susceptible to colonization and recurrent infections by opportunistic pathogens such as *Staphylococcus aureus* and *Pseudomonas aeruginosa*. While *S. aureus* infections typically occur early in the life of PwCF, infections can occur across the lifespan. The recent advances in highly effective CFTR modulator therapies (HEMT) have resulted in significant improvements in the quality and quantity of life for PwCF for the approximately 90% of PwCF in the USA, who have access to HEMT. Recent clinical data demonstrate that while the bacterial burden of pulmonary *S. aureus* infections is decreased in PwCF on HEMT, most *S. aureus* infections are not eradicated (1). In addition, there are significant barriers that prevent widespread access to HEMT for the global CF population. Therefore, there is a long-term need for therapeutic approaches to combat *S. aureus* infections globally in PwCF.

PwCF experiencing pulmonary exacerbation and respiratory infection often require multi-week inpatient hospital care and prolonged antibiotic treatment. However, treating *S. aureus* respiratory infections can be especially problematic due to the high prevalence of antibiotic-resistant strains, such as methicillin-resistant *S. aureus* (MRSA). In addition to these complications in PwCF, *S. aureus* infections cause over 1 million deaths globally each year (2). Thus, novel strategies to combat drug-resistant *S. aureus* infections and inflammation will have a broad impact beyond care for PwCF. One potential therapeutic strategy to combat respiratory infections and inflammation has been developed from understanding the functional roles of mammalian serine threonine protein kinases *Map2k1* (MEK1) and *Map2k2* (MEK2). These two kinases are key regulators of epithelial and immune cell responses to inflammatory stimuli, and activation of this pathway contributes to pro-inflammatory cytokine secretion in CF cell models (3, 4) and human and mouse lung injury (5, 6). We have discovered unique divergent roles of MEK1 and MEK2 in regulating TLR4-mediated inflammation, demonstrating that MEK1 restrains, while MEK2 promotes inflammation, a paradigm we hypothesize extends to additional TLR-mediated inflammatory pathways (5, 6). Further studies have also uncovered important roles of the MEK1/2 pathway in regulating cellular responses of the adaptive immune system, demonstrating that MEK1 and MEK2 have non-redundant and critical roles in regulating B and T cell activation (7). In summary, these studies have identified novel functions of MEK1 and MEK2 that may impact the design of therapeutic strategies to modulate the immune system in multiple disease states and indicate that there are still significant gaps in knowledge regarding the roles of MEK1 and MEK2 in regulating the host response to pathogen infections.

Human and murine MEK1 and MEK2 proteins have amino acid similarity with *S. aureus* Stk1. Stk1 is a eukaryotic-like serine threonine protein kinase that functions in concert with a phosphatase, Stp1 (8). The Stk1-Stp1 pathway regulates multiple pathways in *S. aureus*, with several examples, including cell-wall metabolism by the GraSR two-component system (9) and heme homeostasis (10). The generation of a Stk1-targeting inhibitor compound was demonstrated to decrease MRSA growth and enhance sensitivity to cephalosporins (11). Importantly, a recent study also discovered that the mammalian-targeting MEK1/2 inhibitor compound ATR-002 reduced the growth of *S. aureus*, likely through targeting the Stk1-Stp1 pathway (8). As our prior work (12–14) and that of others (15–18) have demonstrated that MEK1/2 inhibitor compounds can provide beneficial effects in murine models of infection and inflammation, we sought to determine whether the MEK1/2 inhibitor compound ATR-002 elicited dual anti-inflammatory and antibacterial effects that may be harnessed to combat cystic fibrosis *S. aureus* infections.

In this study, *S. aureus* clinical isolates obtained from sputum and sinus from PwCF were utilized to define the antibacterial effects of the MEK1/2 inhibitor compound ATR-002. We found that ATR-002 can decrease TLR2-induced cytokine secretion in human monocyte-derived macrophages from PwCF without impairing macrophage phagocytic abilities. We utilized a murine pulmonary infection model with MRSA strain USA300 to demonstrate the combined anti-inflammatory and antibacterial effects of the MEK1/2 inhibitor compound ATR-002 *in vivo*. In summary, this study highlights the unique ability of a MEK1/2 inhibitor compound to reduce detrimental inflammation and restrict bacterial growth, suggesting ATR-002 may serve as a greatly needed compound to treat antibiotic-resistant *S. aureus* infection.

## MATERIALS AND METHODS

### Reagents

Table S1 contains a list of reagents and their sources used in this study.

### Human monocyte-derived macrophage isolation, culture, and treatment

Donors were consented by clinical research coordinators in the Translational and Data Core of the Cure CF Columbus Research Development Program. Demographic information of blood donors was provided by the Translational and Data Core upon receipt of the de-identified samples. Peripheral blood was isolated from non-CF healthy controls or PwCF by venous puncture and collected in EDTA-vacutainer tubes. Peripheral blood mononuclear cells (PBMCs) were isolated by Ficoll gradient centrifugation methods as previously described (12, 19). Purified PBMCs were incubated in Teflon jars at a concentration of 2 × 10^6^ cells/mL in RPMI-1640 containing 5 mM HEPES, 5 mM L-glutamine, 10% heat-inactivated FBS, and penicillin/streptomycin containing 20 ng/mL of recombinant human M-CSF. Following this incubation, differentiated monocyte-derived macrophages (MDMs) were plated at 300,000 MDMs per well in 24-well tissue culture plates and allowed to adhere to the wells overnight. Cells were rinsed twice in PBS to remove non-adherent cells and then stimulated in media containing 100 ng/mL FSL1 or 1 µg/mL Pam3CSK4 with the addition of either vehicle or ATR-002. Protein lysates and supernatants were collected after 4 hours of stimulation. For phagocytosis experiments, pHrodo Red *S. aureus* bioparticles were prepared following the manufacturer’s recommendations. Particles were mixed with human AB serum following resuspension and incubated for 30 minutes at 37°C with routine mixing to opsonize the *S. aureus* bioparticles. Following opsonization, *S. aureus* bioparticles were added to MDM at a concentration of 0.08 mg/mL for 1 hour at 37°C in media containing either 2 µM cytochalasin-D, vehicle (DMSO), or 200 µM ATR-002. After 1 hour of incubation, MDMs were rinsed to remove uningested particles, incubated in PBS containing 2 mM EDTA, and then removed from tissue culture plates with a cell lifter. MDMs were washed in cell staining buffer, and data were collected on a BD LSRFortessa flow cytometer. Analyses were performed with FlowJo software.

#### *S. aureus* growth in a 96-well plate

Frozen stocks of USA300 and CF *S. aureus* strains were grown on TSA at 37°C overnight, and a single colony of *S. aureus* was used to inoculate 5 mL of BHI broth that was incubated overnight at 37°C with shaking at 200 rpm. Overnight culture was diluted and plated into a 96-well tissue-culture flat-bottom plate so that the final concentration of bacteria was 1:50. Wells were treated with BHI, vehicle (DMSO), ATR-002 (5, 25, or 50 µM), or gentamicin (100 µg/mL). The prepared plate was read at an OD_600_ over a 6 hour period in a SpectraMax iD3 microplate reader. The SpectraMax iD3 was set to 37°C, and the plate underwent shaking (low speed) for 60 seconds before the first read and 120 seconds before each subsequent read at an interval of 30 minutes for the duration of the experiment.

#### *S. aureus* minimum inhibitory concentration testing

Frozen stocks of USA300 and CF *S. aureus* strains were grown on TSA at 37°C overnight. Colonies were suspended in sterile PBS, and suspensions were measured in a spectrophotometer at OD_565_ for an absorbance of 0.50 (±0.01). An ATR-002 stock concentration of 1 mg/mL was prepared in cation-adjusted Mueller Hinton Broth 2 (MHBII). Bacteria and ATR-002 were added to a 96-well tissue-culture U-bottom plate using a broth microdilution plating method. In brief, *S. aureus* bacterial suspensions were diluted in MHBII to a concentration of 1:100. Next, 50 µL of MHBII was added to all wells, and the 1 mg/mL stock solution of ATR-002 was prepared so that an addition of 50 µL to the plate, when serially diluted, provided a testing range of 0.125–64 µg/mL. MHBII was added to all wells at a volume of 100 µL, and *S. aureus* strains were added at a volume of 50 µL to each well, excluding the MHBII-only sterility control wells. Up to four strains, in duplicate, were plated for each experiment. Experimental plates were incubated at 37°C for 16–18 hours. After incubation, plates were visually inspected for growth, and minimum inhibitory concentrations (MICs) were determined by comparing the experimental wells to the sterility control; absorbance at OD_600_ was measured in the SpectraMax iD3 to confirm visual readings. Reported MICs are the result of at least two independent experiments. The same procedure was followed when testing MICs in synthetic CF sputum media 1 (SCFM1), although the overall growth observed in SCFM1 was comparatively less than in MHBII, as quantified by OD_600_ measurements or visual assessment.

### Murine *S. aureus* pulmonary infection

Eight- to twelve-week-old male and female mice were used for infections. C57BL/6J mice were purchased from Jackson Laboratories, while *Mek2*^KO^ and wild-type (WT) control mouse colonies (20) were maintained at OSU. For infections, mice were housed in a BSL2 animal facility in cages with corn cob bedding and provided *ad libitum* access to food and water. An overnight culture of methicillin-resistant *S. aureus* strain USA300 grown in BHI broth was sub-cultured to the mid-logarithmic phase, and bacteria were washed twice in sterile PBS and re-suspended in PBS to deliver 1 × 10^7^ colony-forming units (CFU) in a 50 µL inoculum. Mice were anesthetized with ketamine and xylazine and were provided i.p. injection of either vehicle, PD0325901 (20 mg/kg of body weight), or ATR-002 (10 mg/kg of body weight) in sterile PBS immediately prior to intranasal inoculation with *S. aureus*. Groups of mice were sacrificed at 4 and 24 hours (day 1) after infection. Body mass was measured daily over the course of an infection. Homogenates from the left lung were prepared by Precellys bead (catalog # P000912-LYSK1-A) lysis of tissue in 1 mL of sterile PBS. Serial dilutions (10-fold) of lung homogenates were made in sterile PBS and plated on TSA to quantify CFU; CFU were normalized to gram of tissue collected. Some groups of mice received broncho-alveolar lavage (BAL) on day 1 after infection, and total BAL cell counts were performed using trypan blue and counting on a hemocytometer, with differential staining of cytospin preparations by Kwik-Diff used to enumerate neutrophils and macrophages.

### Statistical analyses

The data in this study were subjected to statistical analyses performed with GraphPad Prism 10 version 10.4.0. The specific statistical tests are listed in each figure legend, and *P* values are reported in the figures.

## RESULTS

### Antibacterial effects of the MEK1/2 inhibitor ATR-002 on *S. aureus* CF clinical isolates

**Fig 1:**
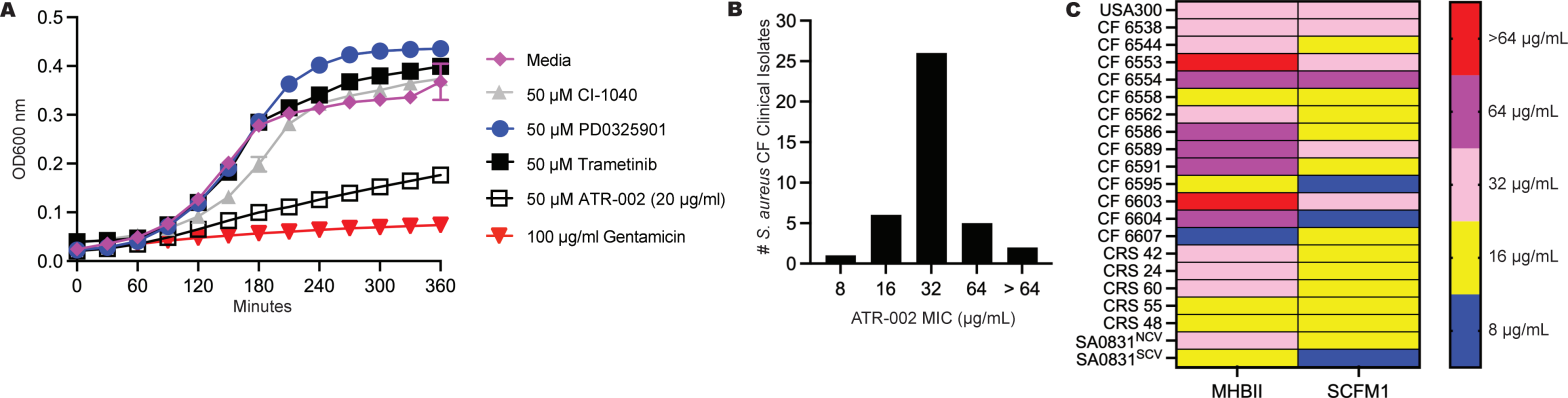
Antibacterial effects of ATR-002 on *S. aureus* USA300 and CF clinical isolates. (**A**) The direct antibacterial effects of the MEK1/2 inhibitor compounds CI-1040, PD0325901, trametinib, or ATR-002 on *S. aureus* strain USA300 (USA300 MIC is 32 µg/mL). Data shown are the mean ± SEM of technical replicates from one representative experiment of three performed in BHI broth. (**B**) Broth microdilution in MHBII was used to quantify the MIC of ATR-002 on a panel of *n* = 40 different CF clinical isolates of *S. aureus* (*n* = 35 isolates from sputum and *n* = 5 isolates from sinus). (**C**) MIC values for some strains were calculated in both MHBII and SCFM1. MIC values were confirmed in at least two independent experiments.

It was previously reported with *in vitro* models that the MEK1/2 inhibitor compound ATR-002 elicited direct antibacterial effects on *S. aureus* (8), while in separate studies, we uncovered the anti-inflammatory effects of several other MEK1/2 inhibitor compounds, including CI-1040, PD0325901, and trametinib (12). In our study, we demonstrated anti-inflammatory effects in human cell culture and murine infection models; however, we did not directly evaluate the potential antibacterial effects of PD0325901 in *S. aureus* or *P. aeruginosa* pulmonary infection models in mice (12, 13). Therefore, to directly compare the potential direct antibacterial effects of MEK1/2 inhibitor compounds on *S. aureus,* the MRSA strain USA300 was inoculated in a 96-well plate in media containing vehicle or 50 µM of PD0325901, CI-1040, trametinib, or ATR-002 and incubated at 37°C with OD_600_ measurements collected over 6 hours. While the addition of gentamicin restricted growth compared to media (Fig. 1A), ATR-002 treatment had a bacteriostatic effect, while other MEK1/2 inhibitor compounds had little effect on restricting bacterial growth (Fig. 1A) (8). To determine if the antibacterial properties of ATR-002 had relevance to CF infections, we quantified the MIC using a microdilution method in MHBII media on a panel of 40 *S*. *aureus* clinical isolates obtained from PwCF. This collection of 40 strains included *S. aureus* isolates from both sputum and nasal collections (see Table 1 for strain information). We discovered that *S. aureus* clinical isolates have a range of susceptibility to ATR-002, with a median MIC of 32 µg/mL (Fig. 1B), and the USA300 strain also had an MIC of 32 µg/mL (Table 1 and Fig. 1C). As *S. aureus* small colony variants (SCVs) are relatively common and associated with reduced lung function and increased risk of pulmonary exacerbations in children with CF (21, 22), we also quantified the MIC of ATR-002 on a CF *S. aureus* normal colony variant (SA0831^NCV^) strain and an isogenic small colony variant of this strain (SA0831^SCV^) and discovered that the SA081^SCV^ strain had an MIC of 16 µg/mL, while the NCV was 32 µg/mL (Table 1 and Fig. 1C), suggesting that ATR-002 could also be utilized as a therapy against *S. aureus* SCV. Finally, as recent literature has demonstrated that bacterial strains may have different susceptibilities to antimicrobial agents due to the *in vivo* host environment (23), we used synthetic media that better model the *in vivo* sputum conditions of PWCF (24) to assess if the MIC of ATR-002 was impacted. We selected *n* = 21 strains across the range of MICs in MHBII for ATR-002 MIC testing in SCFM1 (24), and the results are shown in Fig. 1C. While the MIC of some strains (*n* = 6) remained unchanged between MHBII and SCFM1 media, the majority of isolates tested (*n* = 14) had decreased ATR-002 MIC values in SCFM1 compared to MHBII (Fig. 1C). In contrast, one isolate of those tested, 6607, increased from 8 to 16 µg/mL in SCFM1 media. In summary, these results indicate that ATR-002 appears unique compared to other MEK1/2 inhibitor compounds in eliciting direct antibacterial effects and overall demonstrate that a diverse collection of *S. aureus* clinical isolates is sensitive to growth inhibition by ATR-002, which may be enhanced with conditions that better model *in vivo* conditions.

**TABLE 1:** Strains used in this study

Strains	Description	ATR-002 MIC in MHBII (ATR-002 MIC in SCFM1) (µg/mL)	Reference
Laboratory strain			
*S. aureus* USA300	WT *S. aureus*	32 (32)	(25)
*S. aureus* clinical isolates			
6538	CF sputum clinical isolate	32 (32)	(26)
6539	CF sputum clinical isolate	32	(26)
6540	CF sputum clinical isolate	32	(26)
6541	CF sputum clinical isolate	32	(26)
6542	CF sputum clinical isolate	32	(26)
6543	CF sputum clinical isolate	16	(26)
6544	CF sputum clinical isolate	32 (16)	(26)
6545	CF sputum clinical isolate	16	(26)
6553	CF sputum clinical isolate	>64 (32)	(26)
6554	CF sputum clinical isolate	64 (64)	(26)
6556	CF sputum clinical isolate	32	(26)
6557	CF sputum clinical isolate	32	(26)
6558	CF sputum clinical isolate	16 (16)	(26)
6562	CF sputum clinical isolate	32 (16)	(26)
6563	CF sputum clinical isolate	32	(26)
6564	CF sputum clinical isolate	32	(26)
6567	CF sputum clinical isolate	32	(26)
6569	CF sputum clinical isolate	32	(26)
6586	CF sputum clinical isolate	64 (16)	(26)
6587	CF sputum clinical isolate	32	(26)
6588	CF sputum clinical isolate	32	(26)
6589	CF sputum clinical isolate	64 (32)	(26)
6590	CF sputum clinical isolate	32	(26)
6591	CF sputum clinical isolate	64 (16)	(26)
6592	CF sputum clinical isolate	32	(26)
6593	CF sputum clinical isolate	32	(26)
6594	CF sputum clinical isolate	32	(26)
6595	CF sputum clinical isolate	16 (8)	(26)
6596	CF sputum clinical isolate	32	(26)
6602	CF sputum clinical isolate	32	This study
6603	CF sputum clinical isolate	>64 (32)	This study
6604	CF sputum clinical isolate	64 (8)	This study
6605	CF sputum clinical isolate	32	This study
6606	CF sputum clinical isolate	32	This study
6607	CF sinus clinical isolate	8 (16)	This study
CRS42	CF sinus clinical isolate	32 (16)	(27, 28)
CRS24	CF sinus clinical isolate	32 (16)	(27, 28)
CRS60	CF sinus clinical isolate	32 (16)	(27, 28)
CRS55	CF sinus clinical isolate	16 (16)	(27)
CRS48	CF sinus clinical isolate	16 (16)	(27)
SA0831^NCV^	Parental strain	32 (16)	(29)
SA0831^SCV^	Parental strain	16 (8)	(29)

### ATR-002 reduces CF macrophage TLR2-induced cytokine secretion

**Fig 2:**
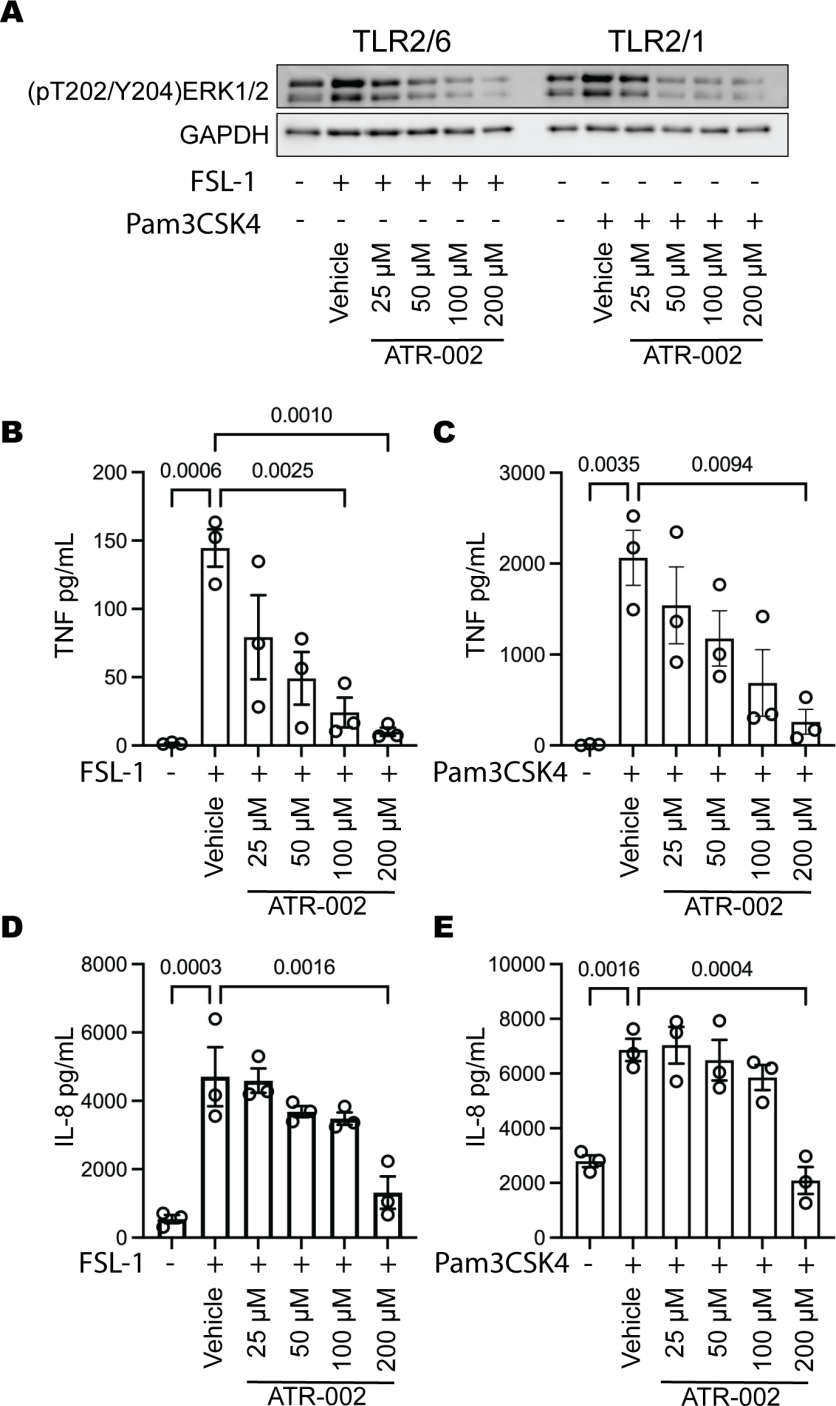
*In vitro* dose-response evaluating the anti-inflammatory effect of ATR-002 on CF macrophages. Monocyte-derived macrophages from people with cystic fibrosis were stimulated with 100 ng/mL FSL1 or 1 µg/mL Pam3CSK4 with either vehicle (DMSO) or increasing doses of ATR-002 added together with TLR agonists to stimulation media, and cells were incubated for 4 hours prior to the collection of cell lysates and supernatants. (**A**) Protein lysates were used in western blots for phosphorylated T202/Y204 ERK1/2 (pT202/Y204 ERK1/2) and GAPDH. Western blot image is one representative experiment of three. Supernatants were used to measure TNF (**B and C**) and IL-8 (**D and E**) by ELISA. Statistical analysis used one-way ANOVA with Tukey’s multiple comparisons. (**B and C**) Data points are cells from three distinct donors, and bars represent the mean ± SEM.

We previously demonstrated that the MEK1/2 inhibitor compounds CI-1040, PD0325901, and trametinib reduced human CF macrophage TLR2/1, TLR2/6, and TLR4-dependent pro-inflammatory cytokine production when added during stimulations with TLR agonists (12). To determine whether ATR-002 elicited similar anti-inflammatory effects and define an effective dose range of ATR-002, monocyte-derived macrophages from PwCF were stimulated for 4 hours with the TLR2/6 agonist FSL1 or the TLR2/1 agonist Pam3CSK4 in media containing either vehicle or an increasing dose of ATR-002. We first demonstrate the ability of ATR-002 to decrease human macrophage MEK1/2-ERK1/2 pathway activation by examining the phosphorylation of ERK1/2 T202/Y204 in CF MDM protein lysates after 4 hours of stimulation (Fig. 2A). We next evaluated TLR-induced TNF and IL-8 cytokine secretion as an endpoint to assess the effects of ATR-002 on macrophage function. While TNF has generally not been associated with clinical outcomes in CF, IL-8-dependent neutrophil recruitment significantly contributes to CF lung disease (30). The levels of TNF (Fig. 2B and C) and IL-8 (Fig. 2D and E) secreted in cell supernatants were examined after 4 hours of stimulation. CF MDM had increased TNF and IL-8 secretion following FSL-1 and Pam3CSK4 stimulation that was significantly reduced by the addition of increasing doses of ATR-002 (Fig. 2B through E). We also performed several experiments analyzing the effects of ATR-002 during FSL-1 stimulation in macrophages from both non-CF healthy controls and PwCF, finding that ATR-002 had similar effects in reducing phosphorylation of ERK1/2 in non-CF MDM when compared to MDM from PwCF (Fig. S1A and B). Overall, while these data indicate potential differential sensitivity in the ability to reduce macrophage pro-inflammatory cytokine secretion, the data confirm the anti-inflammatory potential of ATR-002 on macrophages from non-CF healthy controls or PwCF.

### The MEK1/2 inhibitor ATR-002 does not inhibit CF macrophage phagocytosis

**Fig 3:**
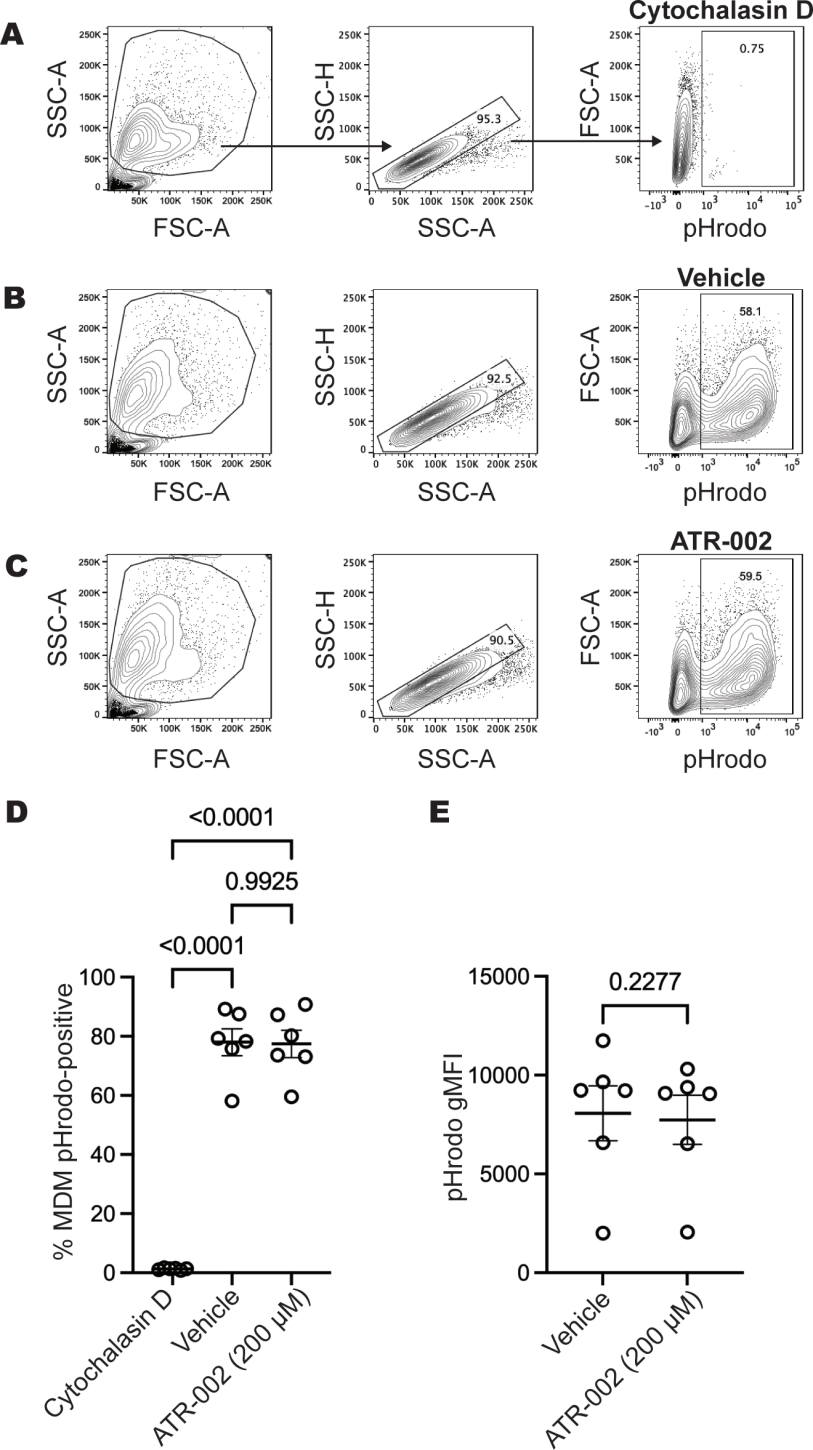
ATR-002 does not prevent phagocytosis or phagosome acidification. Human monocyte-derived macrophages from people with cystic fibrosis were incubated with serum-opsonized *S. aureus* pHrodo-red bioparticles for 1 hour, and phagocytosis was analyzed by flow cytometry. Gating strategy depicts that (**A**) cytochalasin-D-treated MDMs were used to set the pHrodo negative gate for (**B**) vehicle-treated or (**C**) ATR-002-treated samples. (**D**) The percentage of MDM pHrodo-positive and (**E**) geometric mean fluorescence intensity (gMFI) of pHrodo for pHrodo-positive MDMs. Each datapoint represents MDM from a unique donor, and data are combined from two independent experiments. Statistical analysis was performed by (**D**) one-way ANOVA with Tukey’s multiple comparison or (**E**) a paired *t*-test comparing vehicle and ATR-002 groups.

While our previous work demonstrated that the MEK1/2 inhibitor compounds PD0325901, CI-1040, or trametinib did not inhibit or reduce human or murine macrophage phagocytosis abilities (12), the potential effects of ATR-002 on the phagocytic abilities of human macrophages have not been assessed. We utilized serum-opsonized pHrodo red *S. aureus* bioparticles to evaluate whether ATR-002 altered CF macrophage phagocytosis. CF MDM treated with cytochalasin-D were used as a control to inhibit phagocytosis of particles (Fig. 3A). CF MDM treated with vehicle or 200 µM ATR-002 exhibited robust phagocytosis of pHrodo *S. aureus* (Fig. 3B and C), without significant differences in the overall percentage of CF MDM that were pHrodo-red positive between groups (Fig. 3D). In addition, quantification of the geometric mean fluorescent intensity of pHrodo revealed no significant differences between vehicle and ATR-002-treated MDMs (Fig. 3E), indicating that phagosome acidification is not impacted by ATR-002 treatment. Preliminary experiments comparing non-CF MDM with CF MDM did not reveal differential responses to ATR-002 treatment (Fig. S2). Combined, the data here utilizing human CF MDM support the role of the MEK1/2 pathway in regulating macrophage pro-inflammatory cytokine responses to inflammatory agonists but not phagocytic abilities.

### The MEK1/2 inhibitor ATR-002 elicits *in vivo* anti-inflammatory and anti-bacterial effects

**Fig 4:**
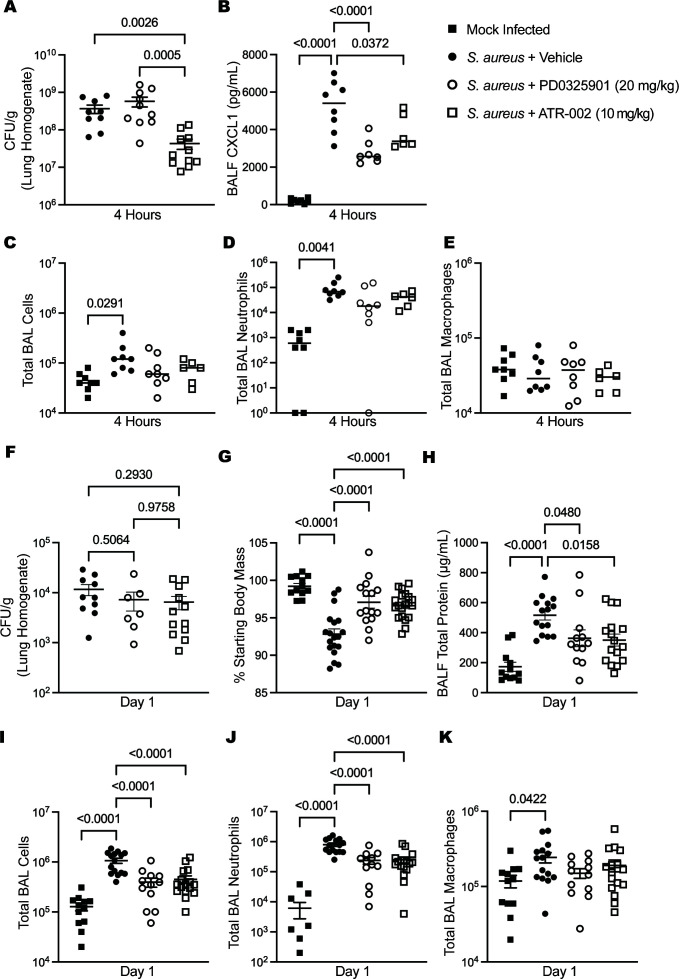
ATR-002 elicits *in vivo* anti-inflammatory and antibacterial effects. Wild-type C57BL6/J mice mock-infected (intranasal delivery of sterile PBS) or treated with i.p. injection of vehicle, 20 mg PD0325901/kg of body weight, or 10 mg ATR-002/kg of body weight and then infected with 1 × 10^7^ CFU of *S. aureus* USA300 by intranasal instillation. At 4 hours after infection, (**A**) bacterial burdens were enumerated from left lung homogenates, and colony-forming units were normalized for gram of tissue collected. BAL was performed at 4 hours after infection, and (**B**) CXCL1 was measured in BAL fluid. At 4 hours after infection, the (**C**) total BAL cells, (**D**) total BAL neutrophils, and (**E**) total BAL macrophages were quantified. On day 1 after infection, (**G**) the percentage of starting body mass, (**H**) BALF total protein, (**I**) total BAL cells, (**J**) total BAL neutrophils, and (**K**) total BAL macrophages were quantified. All data points depict an individual mouse from a minimum of *n* = 2 independent experiments. Data include male and female animals, and bars represent the mean ± SEM. Statistical comparisons used one-way ANOVA with Tukey’s multiple comparisons tests (**B through K**). Data in panel **A** did not have a Gaussian distribution; therefore, a Kruskal-Wallis with Dunn’s multiple comparisons test was used for statistical comparisons.

Based on these *in vitro* antibacterial and anti-inflammatory effects of ATR-002, we hypothesized that *in vivo* administration of ATR-002 would reduce both illness and bacterial burdens in a murine *S. aureus* pulmonary infection model. Our previous work in a pulmonary infection model using the MRSA strain USA300 demonstrated that treatment of mice with the MEK1/2 inhibitor PD0325901 reduced inflammation and illness in a pulmonary infection model without impairing host defense but did not lead to enhanced bacterial clearance, thus indicating PD0325901 had no direct antibacterial effect on *S. aureus* (12). While CF mouse models exist, they do not recapitulate the inflammatory milieu and bacterial infections in human CF. In addition, although chronic bacterial infections are common in human CF, new acute infections, including with *S. aureus,* still occur, and therefore, the development of anti-inflammatory and antimicrobial therapies to treat new infections or pulmonary exacerbations in CF has clinical relevance. To model an acute *S. aureus* infection, we performed a comparative study using C57BL6/J male and female mock-infected and infected mice treated with vehicle, 20 mg PD0325901/kg of body weight, or 10 mg ATR-002/kg of body weight to test the hypothesis that ATR-002 could reduce bacterial burdens and decrease inflammation during *in vivo S. aureus* pulmonary infection. Endpoint measurements were examined at 4 hours and 1 day after infection. The dose of 10 mg ATR-002/kg of body weight was selected as this is within the dose range provided in a recent COVID-19 clinical trial (31), while 20 mg/kg of body weight was used for consistency with our previous studies (12–14). To examine the effects of MEK1/2 inhibitor treatments on bacterial burdens, quantification of CFU from lung homogenates at 4 hours post-infection revealed that the ATR-002-treated group had a significant reduction in bacterial burdens compared to vehicle or PD0325901-treated groups (Fig. 4A). BAL was also performed at 4 hours, and the level of CXCL1 was significantly reduced in both PD0325901- and ATR-002-treated groups compared to vehicle treatment (Fig. 4B), while only PD0325901 treatment significantly reduced BAL fluid (BALF) TNF (Fig. S3). The total BAL cells (Fig. 4C) and BAL neutrophils (Fig. 4D) were significantly increased in the *S. aureus* vehicle-treated group compared to mock infection; however, infected groups treated with either PD0325901 or ATR-002 were not significantly different from mock-infected or the *S. aureus* vehicle-treated groups, indicating a potential moderate anti-inflammatory effect from MEK1/2 inhibitor treatments at this early time point. There were no significant changes in the levels of BAL macrophages between any groups (Fig. 4E). On day 1 after infection, CFU in lung homogenates were no longer significantly different between groups (Fig. 4E); however, overall bacterial clearance on day 1 after infection is high in this model of infection, demonstrating robust clearance that is not impaired by either MEK1/2 inhibitor treatment. In contrast, the infection induced a significant reduction in body mass on day 1, an indicator of illness, compared to mock infection, which was abrogated by PD0325901 and ATR-002 treatment (Fig. 4G). BALF total protein was also significantly reduced by PD0325901 and ATR-002 treatments (Fig. 4H). The reduction in illness and lung injury in the MEK1/2 inhibitor-treated groups corresponded with a significant reduction in the recruitment of inflammatory cells to the alveolar spaces, as the total BAL cells (Fig. 4I) and total BAL neutrophils (Fig. 4J) in PD0325901- and ATR-002-treated mice were significantly reduced compared to vehicle-treated mice. Highlighting the marked reduction in neutrophils, the mean neutrophil levels of the PD0325901 and ATR-002 groups were 29.6% and 31.4% of the vehicle group, respectively. While there was a modest increase in the total BAL macrophage numbers in infected vehicle-treated mice compared to mock-infected mice, treatment of infected mice with PD0325901 or ATR-002 did not significantly alter macrophage levels (Fig. 4K). Overall, these data support the hypothesis that ATR-002 elicits beneficial antibacterial and anti-inflammatory effects during *in vivo S. aureus* infection and demonstrate a robust *in vivo* antibacterial effect of ATR-002 treatment specifically.

### MEK2 deletion reduces inflammation during *S. aureus* pulmonary infection

**Fig 5:**
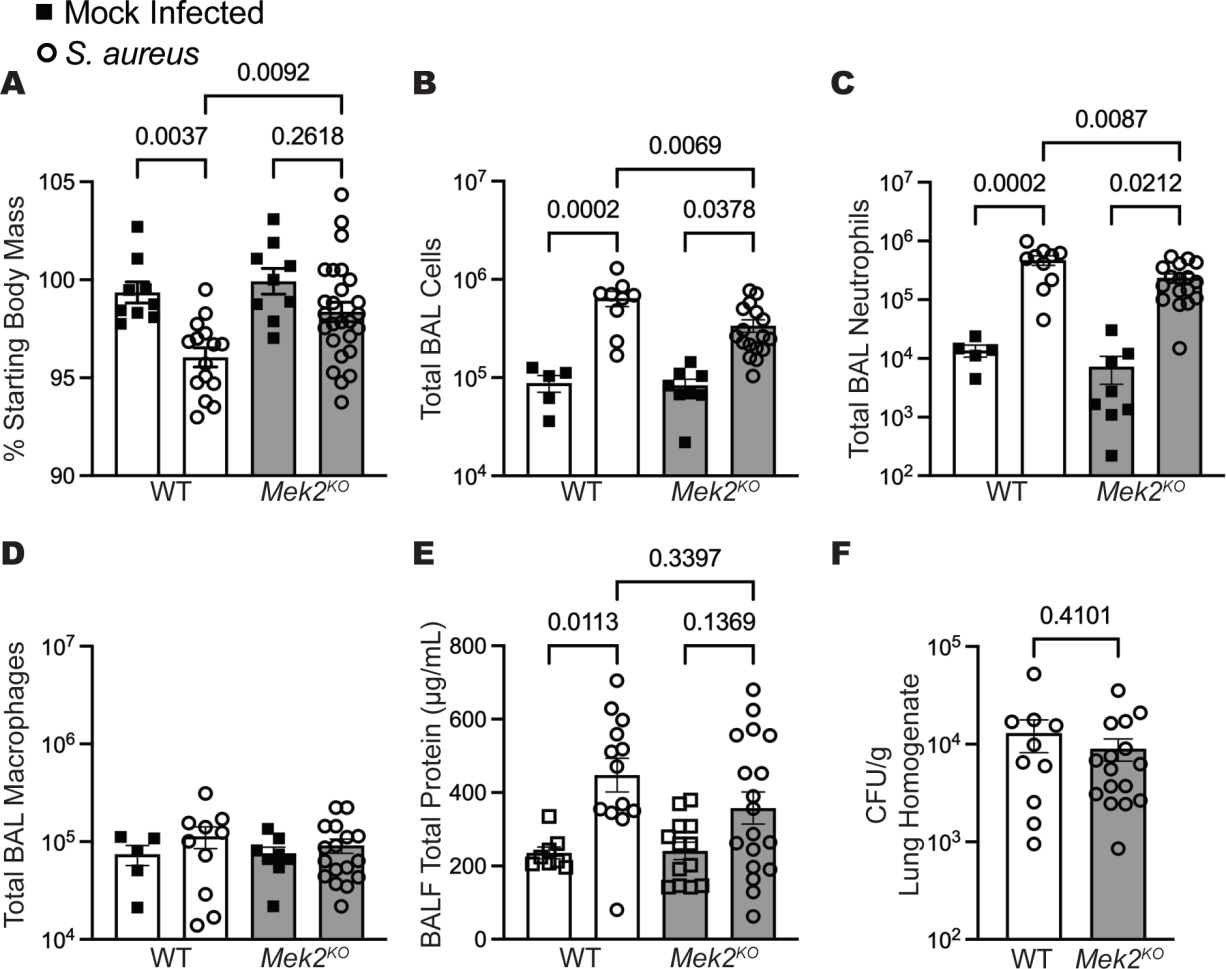
MEK2 deletion reduces *S. aureus* infection-induced inflammation. Wild-type or *Mek2^KO^* mice were mock-infected (intranasal delivery of sterile PBS) or infected with 1 × 10^7^ CFU of *S. aureus* USA300 by intranasal instillation. On day 1 after infection, (A) the percentage of starting body mass, (B) total BAL cells, (C) total BAL neutrophils, and (D) total BAL macrophages were quantified by cell counts and differential staining on cytospin preparation, and (E) total protein from BALF was quantified. (F) Bacterial burdens were enumerated from left lung homogenates prepared on day 1 after infection. CFU were normalized for the gram of tissue collected. All datapoints depict an individual mouse from a minimum of *n* = 3 (A–F) independent experiments. Data include male and female animals, and bars represent the mean ± SEM. Statistical comparisons used one-way ANOVA with Tukey’s multiple comparisons tests (A through D) or a Student’s *t*-test (F).

Given the dual targeting of MEK1 and MEK2 by ATR-002, we wanted to further understand the mechanisms of how inhibition of these kinases regulates host inflammation. Our previous results demonstrated that MEK2 was a driver of detrimental pro-inflammatory responses during LPS-induced acute lung injury or *P. aeruginosa* infection (6). However, whether MEK2 has a similar deleterious role during gram-positive bacterial infections is not known. We predicted that MEK2 activation may be contributing to deleterious inflammation following *S. aureus* infection. To investigate the role of MEK2 during *S. aureus* infection, wild-type or *Mek2*^KO^ mice were mock-infected or infected with 1 × 10^7^ CFU *S*. *aureus* strain USA300 by intranasal instillation. Compared to WT, *Mek2*^KO^ mice showed significant preservation in body mass on day 1 after infection (Fig. 5A) and a decreased total number of BAL cells (Fig. 5B), which was predominately due to a reduction in the total number of BAL neutrophils (Fig. 5C), without changes in the total number of BAL macrophages (Fig. 5D). While BALF total protein was significantly increased in WT mice at 24 hours following infection (Fig. 5E), consistent with elevated alveolar damage or leak, *Mek2^KO^* mice had an intermediate phenotype where infection resulted in a non-significant increase compared to mock infection and a non-significant decrease compared to infected WT mice. Importantly, *Mek2*^KO^ mice did not have impaired bacterial clearance, as the bacterial burdens in the lung were similar to WT mice (Fig. 5F). This suggests that MEK2 promotes inflammation but is dispensable for the regulation of *S. aureus* bacterial burden. However, whether ATR-002 would still elicit an antibacterial effect in *Mek2^KO^*, where inflammation is significantly reduced, was unclear. To determine whether the MEK1/2 inhibitor ATR-002 would elicit an antibacterial effect in *Mek2*^KO^ mice infected with USA300, bacterial burdens in the lung at 4 hours of infection in *Mek2*^KO^ mice treated with either vehicle or ATR-002 were quantified, revealing that *Mek2*^KO^ mice treated with ATR-002 also had a significant reduction in bacterial burden (Fig. S4). In summary, while the deletion of host MEK2 reduces the inflammatory response to *S. aureus* infection without impairing host defense, ATR-002 treatment is able to further reduce pathogen burden after infection by its direct antibacterial effects.

## DISCUSSION

The rise and global expansion of multidrug-resistant *S. aureus* requires the development of new therapeutic approaches to treat these challenging infections (32). As the excessive host inflammatory response is often a significant contributor to tissue pathology and disease, especially during pulmonary infections (33), therapeutic strategies targeted to limit host inflammation and immunopathology are promising. Investigations into the antiviral properties of MEK1/2 inhibitor compounds (34, 35) recently culminated in a human clinical trial of ATR-002 treatment during COVID-19 infection. The trial was terminated early due to low recruitment, which was impacted by the Omicron variant and less severe disease (31). However, ATR-002 may have further therapeutic utility for treating other respiratory viral infections or respiratory viral and bacterial co-infections. PwCF encounter chronic and recurrent *S. aureus* pulmonary infections, and CF pulmonary exacerbations are often associated with a respiratory viral infection (36). Therefore, the antimicrobial and anti-inflammatory therapeutic potential of ATR-002 may have multifactorial beneficial therapeutic effects in the context of CF pulmonary infections. However, the ability of ATR-002 to elicit anti-inflammatory effects on CF cells or antibacterial effects on clinically relevant CF *S. aureus* strains has not been investigated. Furthermore, the *in vivo* antibacterial and anti-inflammatory effects of ATR-002 have not been investigated in the context of an *S. aureus* infection model.

In this study, we investigated the *in vitro* anti-inflammatory and antibacterial effects of the MEK1/2 inhibitor compound ATR-002 utilizing human CF macrophages and clinical isolates of *S. aureus*. Using a collection of CF *S. aureus* clinical isolates, a range of *S. aureus* susceptibility to the MEK1/2 inhibitor compound ATR-002 was discovered with a median MIC of 32 µg/mL, which was the same MIC value for the community-associated MRSA strain USA300. The *S. aureus* strains isolated from CF sputum were predominantly from adults and likely reflect strains in individuals with more advanced disease; it is not known whether *S. aureus* infections that occur within the first several years of life in PwCF would exhibit similar sensitivities to ATR-002, although we suspect they would. While these results were obtained in MHBII media, a standard for antimicrobial testing, it has been reported that refined *in vitro* media likely do not represent the complex *in vivo* host environment. Therefore, while MHBII media provides a good reference standard, it may not accurately depict treatment responses. In the context of CF pulmonary infections, it has been recognized that bacterial responses in standard media also likely do not reflect the *in vivo* host environment (37, 38); therefore, the development of synthetic sputum medium has been used to help better model the *in vivo* CF environment (24). Quantification of ATR-002 MICs in SCFM1 media generally resulted in a lower MIC compared to MHBII, although there were some strains without changes and one strain that had a higher MIC. Overall, these data suggest that ATR-002 may have increased antimicrobial effects in the context of CF pulmonary infection. Furthermore, a small colony variant of a CF isolate (29) remained susceptible to ATR-002 and even had a lower MIC value of 16 µg/mL compared to its normal colony variant parental strain, which had an MIC of 32 µg/mL. This finding suggests that ATR-002 may have utility in targeting a broad range of clinically relevant *S. aureus* infections, including small colony variants that are associated with increased inflammation and worse outcomes in PwCF (21, 29). While our studies evaluated both nasal and sputum CF clinical isolates, we did not observe overtly different effects of ATR-002 on these strains, though the sample sizes for this study remain relatively small. Importantly, ATR-002 treatment significantly decreased lung bacterial burdens in a murine MRSA pulmonary infection model at 4 hours, providing the first *in vivo* demonstration of the antibacterial effects of this compound on *S. aureus*. While the *in vitro* data suggest that ATR-002 is bacteriostatic, potentially limiting its use as an antibiotic, it remains to be determined whether ATR-002 alters *S. aureus* sensitivity to antibiotics or immune defense mechanisms and antimicrobial proteins. Previous studies demonstrated that ATR-002 increased *S. aureus* sensitivity to gentamicin (8), though the clinical utility of this is limited. Interestingly, a recent report also demonstrated that combined treatment of gentamicin with the MEK1/2 inhibitor PD0325901 (ATR-002 was not investigated in the study) with administration starting on day 5 post-infection reduced pathology and bacterial burdens in a murine model of *S. aureus* osteomyelitis (39). Results from this study indicated that PD0325901 prevented the *S. aureus*-induced decrease in mitochondrial reactive oxygen species observed 12 hours after the infection of macrophages, which was suggested to enhance the microbicidal capacity of macrophages (39). As our data here did not detect direct antibacterial effects of PD0325901 or alterations in bacterial clearance in tissues 24 hours after infection (12, 13), different findings could result from different routes of infection or *S. aureus* strains; however, alterations in immune system antimicrobial host defenses during chronic infections should be further investigated.

While MEK1/2 inhibitor compounds target and inhibit both MEK1 and MEK2 proteins, recent studies have demonstrated unique roles of MEK1 and MEK2 in regulating the response to inflammatory stimuli. For example, our previous investigations demonstrated that *Mek1^fl^LysM^Cre^* deletion of *Mek1* exacerbated LPS-ALI (5), while *Mek2^KO^* mice were protected in models of LPS-ALI or acute *P. aeruginosa* pulmonary infection (6). Here, we also demonstrate that *Mek2^KO^* is host protective during MRSA pulmonary infection without impacting bacterial clearance, further suggesting that MEK2-dependent signaling pathways are a driver of host-mediated inflammation. In summary, this study further demonstrates the therapeutic potential of targeting the MEK1/2 pathway using pharmacologic inhibitor compounds that elicit both host-targeted and pathogen-targeted effects.

While this study reduces several gaps in knowledge, there are important limitations that should be considered. First, the mechanism of action for ATR-002 and the basis for ATR-002 resistance in some CF clinical isolates of *S. aureus* are incompletely understood, and it is possible that the microbiologic effect of ATR-002 is transient with a limited therapeutic potential *in vivo*. For example, the bioavailability of ATR-002 and its efficacy in the airways or sputum of PwCF are unknown. Second, the mouse model of *S. aureus* pulmonary infection is a modest lung injury model with relatively rapid clearance of infection despite a high inoculum of 1 × 10^7^ CFU. While there are now a variety of CF mouse models available, these models do not recapitulate spontaneous pulmonary manifestations or infections that occur in human CF (40, 41). Similarly, the wild-type mouse model does not fully recapitulate important host-pathogen interactions in severe or recurrent pulmonary infections, such as those in PwCF. However, it should also be noted that PwCF on CFTR modulator therapies maintain chronic infections and acquire new infections, despite having CFTR function restored (42). This suggests that acute models of infection, even in the presence of functional CFTR, may have relevance for CF disease. How ATR-002 may impact immune cell function in PwCF who are on CFTR modulators was not investigated here but should be further pursued in future studies. Interestingly, previous reports have shown that inhibitors targeting the MEK1/2 pathway may help to restore CFTR function in epithelial cells (4, 43), suggesting there may be additional benefits of MEK1/2 inhibitor therapy during acute infections. A further limitation of the current study is that the transient nature of this murine pulmonary infection model requires the i.p. treatments of MEK1/2 inhibitor compounds to be provided immediately prior to infection, limiting the ability to better model a translational intervention where ATR-002 treatment could be provided subsequent to infection. Given these limitations, future studies should investigate the anti-inflammatory and antibacterial effects of ATR-002 in experimental models such as the CF rat, which has recently been demonstrated to better recapitulate pulmonary manifestations of CF and serve as models of acute and chronic infection or polymicrobial infection (44).

## SUPPLEMENTAL MATERIAL

Supplemental Material mbio.00775-25-s0001.pdf Figures S1 to S4 and Table S1.

ASM does not own the copyrights to Supplemental Material that may be linked to, or accessed through, an article. The authors have granted ASM a non-exclusive, world-wide license to publish the Supplemental Material files. Please contact the corresponding author directly for reuse.

## References

[1] Nichols DP, Morgan SJ, Skalland M, Vo AT, Van Dalfsen JM, Singh SB, Ni W, Hoffman LR, McGeer K, Heltshe SL, Clancy JP, Rowe SM, Jorth P, Singh PK, PROMISE-Micro Study Group. 2023. Pharmacologic improvement of CFTR function rapidly decreases sputum pathogen density, but lung infections generally persist. J Clin Invest 133:e167957. doi:10.1172/JCI167957 36976651 PMC10178839

[2] Antimicrobial Resistance C. 2022. Global burden of bacterial antimicrobial resistance in 2019: a systematic analysis. Lancet 399:629–655. 35065702 10.1016/S0140-6736(21)02724-0 PMC8841637

[3] Bhattacharyya S, Gutti U, Mercado J, Moore C, Pollard HB, Biswas R. 2011. MAPK signaling pathways regulate IL-8 mRNA stability and IL-8 protein expression in cystic fibrosis lung epithelial cell lines. Am J Physiol Lung Cell Mol Physiol 300:L81–7. doi:10.1152/ajplung.00051.2010 20952496 PMC3023294

[4] Xu X, Balsiger R, Tyrrell J, Boyaka PN, Tarran R, Cormet-Boyaka E. 2015. Cigarette smoke exposure reveals a novel role for the MEK/ERK1/2 MAPK pathway in regulation of CFTR. Biochim Biophys Acta 1850:1224–1232. doi:10.1016/j.bbagen.2015.02.004 25697727 PMC4398641

[5] Long ME, Gong K-Q, Eddy WE, Volk JS, Morrell ED, Mikacenic C, West TE, Skerrett SJ, Charron J, Liles WC, Manicone AM. 2019. MEK1 regulates pulmonary macrophage inflammatory responses and resolution of acute lung injury. JCI Insight 4:e132377. doi:10.1172/jci.insight.132377 31801908 PMC6962022

[6] Gong K-Q, Mikacenic C, Long ME, Frevert CW, Birkland TP, Charron J, Gharib SA, Manicone AM. 2022. MAP2K2 Delays recovery in murine models of acute lung injury and associates with acute respiratory distress syndrome outcome. Am J Respir Cell Mol Biol 66:555–563. doi:10.1165/rcmb.2021-0252OC 35157553 PMC9116357

[7] Houde N, Beuret L, Bonaud A, Fortier-Beaulieu S-P, Truchon-Landry K, Aoidi R, Pic É, Alouche N, Rondeau V, Schlecht-Louf G, Balabanian K, Espéli M, Charron J. 2022. Fine-tuning of MEK signaling is pivotal for limiting B and T cell activation. Cell Rep 38:110223. doi:10.1016/j.celrep.2021.110223 35021072

[8] Bruchhagen C, Jarick M, Mewis C, Hertlein T, Niemann S, Ohlsen K, Peters G, Planz O, Ludwig S, Ehrhardt C. 2018. Metabolic conversion of CI-1040 turns a cellular MEK-inhibitor into an antibacterial compound. Sci Rep 8:9114. doi:10.1038/s41598-018-27445-7 29904167 PMC6002397

[9] Fridman M, Williams GD, Muzamal U, Hunter H, Siu KWM, Golemi-Kotra D. 2013. Two unique phosphorylation-driven signaling pathways crosstalk in staphylococcus aureus to modulate the cell-wall charge: Stk1/Stp1 meets GraSR. Biochemistry 52:7975–7986 (2013). doi:10.1021/bi401177n 24102310

[10] Leasure CS, Grunenwald CM, Choby JE, Sauer JD, Skaar EP. 2023. Maintenance of heme homeostasis in Staphylococcus aureus through post-translational regulation of glutamyl-tRNA reductase. J Bacteriol 205:e00171-23. doi:10.1128/jb.00171-23 37655914 PMC10521356

[11] Kant S, Asthana S, Missiakas D, Pancholi V. 2017. A novel STK1-targeted small-molecule as an “antibiotic resistance breaker” against multidrug-resistant Staphylococcus aureus. Sci Rep 7:5067. doi:10.1038/s41598-017-05314-z 28698584 PMC5505960

[12] De M, Serpa G, Zuiker E, Hisert KB, Liles WC, Manicone AM, Hemann EA, Long ME. 2024. MEK1/2 inhibition decreases pro-inflammatory responses in macrophages from people with cystic fibrosis and mitigates severity of illness in experimental murine methicillin-resistant Staphylococcus aureus infection. Front Cell Infect Microbiol 14:1275940. doi:10.3389/fcimb.2024.1275940 38352056 PMC10861668

[13] Long ME, Gong KQ, Eddy WE, Liles WC, Manicone AM. 2017. Pharmacologic inhibition of MEK1/2 reduces lung inflammation without impairing bacterial clearance in experimental Pseudomonas aeruginosa pneumonia. Pneumonia (Nathan) 9:13. doi:10.1186/s41479-017-0037-y 28879065 PMC5583963

[14] Long ME, Eddy WE, Gong K-Q, Lovelace-Macon LL, McMahan RS, Charron J, Liles WC, Manicone AM. 2017. MEK1/2 Inhibition promotes macrophage reparative properties. J Immunol 198:862–872. doi:10.4049/jimmunol.1601059 28003382 PMC5224968

[15] Smith JA, Mayeux PR, Schnellmann RG. 2016. Delayed mitogen-activated protein kinase/extracellular signal-regulated kinase inhibition by trametinib attenuates systemic inflammatory responses and multiple organ injury in murine sepsis. Crit Care Med 44:e711–20. doi:10.1097/CCM.0000000000001672 27031380 PMC4949133

[16] Schuh K, Pahl A. 2009. Inhibition of the MAP kinase ERK protects from lipopolysaccharide-induced lung injury. Biochem Pharmacol 77:1827–1834. doi:10.1016/j.bcp.2009.03.012 19428337

[17] Droebner K, Pleschka S, Ludwig S, Planz O. 2011. Antiviral activity of the MEK-inhibitor U0126 against pandemic H1N1v and highly pathogenic avian influenza virus in vitro and in vivo. Antiviral Res 92:195–203. doi:10.1016/j.antiviral.2011.08.002 21854809

[18] Wu X, Dayanand KK, Thylur RP, Norbury CC, Gowda DC. 2017. Small molecule-based inhibition of MEK1/2 proteins dampens inflammatory responses to malaria, reduces parasite load, and mitigates pathogenic outcomes. J Biol Chem 292:13615–13634. doi:10.1074/jbc.M116.770313 28679535 PMC5566520

[19] Long ME, Lindemann SR, Rasmussen JA, Jones BD, Allen L-AH. 2013. Disruption of Francisella tularensis Schu S4 iglI, iglJ, and pdpC genes results in attenuation for growth in human macrophages and in vivo virulence in mice and reveals a unique phenotype for pdpC. Infect Immun 81:850–861. doi:10.1128/IAI.00822-12 23275090 PMC3584877

[20] Bélanger L-F, Roy S, Tremblay M, Brott B, Steff A-M, Mourad W, Hugo P, Erikson R, Charron J. 2003. Mek2 is dispensable for mouse growth and development. Mol Cell Biol 23:4778–4787. doi:10.1128/MCB.23.14.4778-4787.2003 12832465 PMC162209

[21] Wolter DJ, Emerson JC, McNamara S, Buccat AM, Qin X, Cochrane E, Houston LS, Rogers GB, Marsh P, Prehar K, Pope CE, Blackledge M, Déziel E, Bruce KD, Ramsey BW, Gibson RL, Burns JL, Hoffman LR. 2013. Staphylococcus aureus small-colony variants are independently associated with worse lung disease in children with cystic fibrosis. Clin Infect Dis 57:384–391. doi:10.1093/cid/cit270 23625938 PMC3888146

[22] Wolter DJ, Onchiri FM, Emerson J, Precit MR, Lee M, McNamara S, Nay L, Blackledge M, Uluer A, Orenstein DM, Mann M, Hoover W, Gibson RL, Burns JL, Hoffman LR, SCVSA study group. 2019. Prevalence and clinical associations of Staphylococcus aureus small-colony variant respiratory infection in children with cystic fibrosis (SCVSA): a multicentre, observational study. Lancet Respir Med 7:1027–1038. doi:10.1016/S2213-2600(19)30365-0 31727592 PMC6924508

[23] Heithoff DM, Barnes V L, Mahan SP, Fried JC, Fitzgibbons LN, House JK, Mahan MJ. 2023. Re-evaluation of FDA-approved antibiotics with increased diagnostic accuracy for assessment of antimicrobial resistance. Cell Rep Med 4:101023. doi:10.1016/j.xcrm.2023.101023 37116500 PMC10213814

[24] Palmer KL, Aye LM, Whiteley M. 2007. Nutritional cues control Pseudomonas aeruginosa multicellular behavior in cystic fibrosis sputum. J Bacteriol 189:8079–8087. doi:10.1128/JB.01138-07 17873029 PMC2168676

[25] Greenlee-Wacker MC, Kremserová S, Nauseef WM. 2017. Lysis of human neutrophils by community-associated methicillin-resistant Staphylococcus aureus. Blood 129:3237–3244 (2017). doi:10.1182/blood-2017-02-766253 28473408 PMC5472901

[26] Liu Y, et al.. 2024. A bacterial pigment provides cross-species protection from H(2)O(2)- and neutrophil-mediated killing. Proc Natl Acad Sci USA 121:e2312334121. doi:10.1073/pnas.2312334121 38170744 PMC10786307

[27] Zemke AC, Nouraie SM, Moore J, Gaston JR, Rowan NR, Pilewski JM, Bomberger JM, Lee SE. 2019. Clinical predictors of cystic fibrosis chronic rhinosinusitis severity. Int Forum Allergy Rhinol 9:759–765. doi:10.1002/alr.22332 31162888 PMC6715283

[28] Huffines JT, Boone RL, Kiedrowski MR. 2024. Temperature influences commensal-pathogen dynamics in a nasal epithelial cell co-culture model. mSphere 9:e00589-23. doi:10.1128/msphere.00589-23 38179905 PMC10826359

[29] Bollar GE, Keith JD, Oden AM, Kiedrowski MR, Birket SE. 2022. Acute infection with a tobramycin-induced small colony variant of Staphylococcus aureus causes increased inflammation in the cystic fibrosis rat lung. Infect Immun 90:e00237-22. doi:10.1128/iai.00237-22 36165627 PMC9671023

[30] Guan X, Hou Y, Sun F, Yang Z, Li C. 2016. Dysregulated chemokine signaling in cystic fibrosis lung disease: a potential therapeutic target. Curr Drug Targets 17:1535–1544. doi:10.2174/1389450117666151209120516 26648071 PMC6500735

[31] Rohde G, Stenglein S, Prozesky H, Manudhane G, Sandulescu O, Bauer M, Overend T, Koch W, Neuschwander D, Planz O, Torres A, Witzenrath M. 2023. Efficacy and safety of zapnometinib in hospitalised adult patients with COVID-19 (RESPIRE): a randomised, double-blind, placebo-controlled, multicentre, proof-of-concept, phase 2 trial. EClinicalMedicine 65:102237. doi:10.1016/j.eclinm.2023.102237 38106555 PMC10725048

[32] De Oliveira DMP, Forde BM, Kidd TJ, Harris PNA, Schembri MA, Beatson SA, Paterson DL, Walker MJ. 2020. Antimicrobial resistance in ESKAPE pathogens. Clin Microbiol Rev 33:e00181-19. doi:10.1128/CMR.00181-19 32404435 PMC7227449

[33] Long ME, Mallampalli RK, Horowitz JC. 2022. Pathogenesis of pneumonia and acute lung injury. Clin Sci (Lond) 136:747–769. doi:10.1042/CS20210879 35621124 PMC9429452

[34] Ludwig S, Wolff T, Ehrhardt C, Wurzer WJ, Reinhardt J, Planz O, Pleschka S. 2004. MEK inhibition impairs influenza B virus propagation without emergence of resistant variants. FEBS Lett 561:37–43 (2004). doi:10.1016/S0014-5793(04)00108-5 15013748

[35] Pleschka S, Wolff T, Ehrhardt C, Hobom G, Planz O, Rapp UR, Ludwig S. 2001. Influenza virus propagation is impaired by inhibition of the Raf/MEK/ERK signalling cascade. Nat Cell Biol 3:301–305. doi:10.1038/35060098 11231581

[36] Wark PAB, Tooze M, Cheese L, Whitehead B, Gibson PG, Wark KF, McDonald VM. 2012. Viral infections trigger exacerbations of cystic fibrosis in adults and children. Eur Respir J 40:510–512. doi:10.1183/09031936.00202311 22855475

[37] Dorschner RA, Lopez-Garcia B, Peschel A, Kraus D, Morikawa K, Nizet V, Gallo RL. 2006. The mammalian ionic environment dictates microbial susceptibility to antimicrobial defense peptides. FASEB J 20:35–42. doi:10.1096/fj.05-4406com 16394265

[38] Lin L, Nonejuie P, Munguia J, Hollands A, Olson J, Dam Q, Kumaraswamy M, Rivera H Jr, Corriden R, Rohde M, Hensler ME, Burkart MD, Pogliano J, Sakoulas G, Nizet V. 2015. Azithromycin synergizes with cationic antimicrobial peptides to exert bactericidal and therapeutic activity against highly multidrug-resistant gram-negative bacterial pathogens. EBioMedicine 2:690–698. doi:10.1016/j.ebiom.2015.05.021 26288841 PMC4534682

[39] Jin M, Wu X, Hu J, Chen Y, Yang B, Cheng C, Yang M, Zhang X. 2024. EGFR-MEK1/2 cascade negatively regulates bactericidal function of bone marrow macrophages in mice with Staphylococcus aureus osteomyelitis. PLoS Pathog 20:e1012437. doi:10.1371/journal.ppat.1012437 39102432 PMC11326603

[40] Shah VS, Meyerholz DK, Tang XX, Reznikov L, Abou Alaiwa M, Ernst SE, Karp PH, Wohlford-Lenane CL, Heilmann KP, Leidinger MR, Allen PD, Zabner J, McCray PB Jr, Ostedgaard LS, Stoltz DA, Randak CO, Welsh MJ. 2016. Airway acidification initiates host defense abnormalities in cystic fibrosis mice. Science 351:503–507. doi:10.1126/science.aad5589 26823428 PMC4852973

[41] McCarron A, Parsons D, Donnelley M. 2021. Animal and cell culture models for cystic fibrosis: which model is right for your application? Am J Pathol 191:228–242. doi:10.1016/j.ajpath.2020.10.017 33232694

[42] Morgan SJ, Coulter E, Betts HL, Solomon GM, Clancy JP, Rowe SM, Nichols DP, Singh PK, PROMISE-Micro Study Group. 2024. Elexacaftor/tezacaftor/ivacaftor’s effects on cystic fibrosis infections are maintained, but not increased, after 3.5 years of treatment. J Clin Invest 134:e184171. doi:10.1172/JCI184171 39235967 PMC11473140

[43] Wellmerling J, Rayner RE, Chang S-W, Kairis EL, Kim SH, Sharma A, Boyaka PN, Cormet-Boyaka E. 2022. Targeting the EGFR-ERK axis using the compatible solute ectoine to stabilize CFTR mutant F508del. FASEB J 36:e22270. doi:10.1096/fj.202100458RRR 35412656 PMC9009300

[44] Bollar GE, Keith JD, Stanford DD, Oden AM, Raju SV, Poore TS, Birket SE. 2025. Chronic coinfection with pseudomonas aeruginosa and normal colony staphylococcus aureus causes lung structural damage in the cystic fibrosis rat. Am J Pathol 195:174–187. doi:10.1016/j.ajpath.2024.09.008 39476957 PMC11773620

