# Supplementary material for: Targeting the MEK1/2 pathway to combat Staphylococcus aureus infection and inflammation in cystic fibrosis
Source: mBio. 2025 May 27;16(7):e00775-25. doi: 10.1128/mbio.00775-25 (PMC12239583; doi:10.1128/mbio.00775-25)
Supplement: Supplemental Material — Figures S1 to S4 and Table S1. [file mbio.00775-25-s0001.pdf]

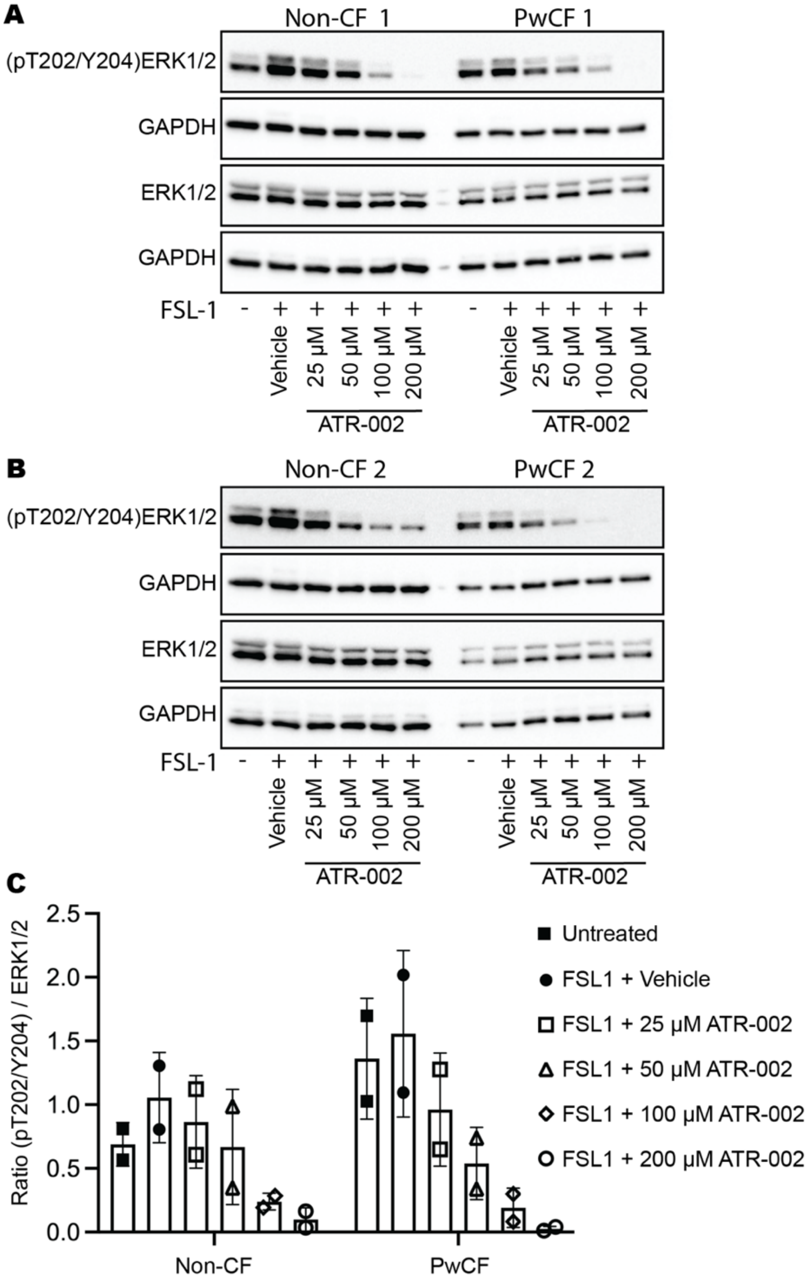

Supplemental Figure 1. Comparison of TLR2/6 Stimulation in Human Monocyte Derived Macrophages from Non-CF Donors and PwCF.

(A-B) Human monocyte-derived macrophages (MDM) from non-CF healthy donors and PwCF were matched for an individual experiment. MDM were either untreated or stimulated with FSL-1 and the addition of vehicle or increasing doses of ATR-002 for 4 hours. Protein lysates were collected and matched samples from Non-CF and PwCF were loaded into the same gel for western blot analyses or phosphorylated ERK1/2 (pERK1/2) and total ERK1/2. Blots were stripped and re-probed for GAPDH. (C) Densitometry quantification of blots was performed using ImageJ to determine the ratio or pERK1/2 to ERK1/2.

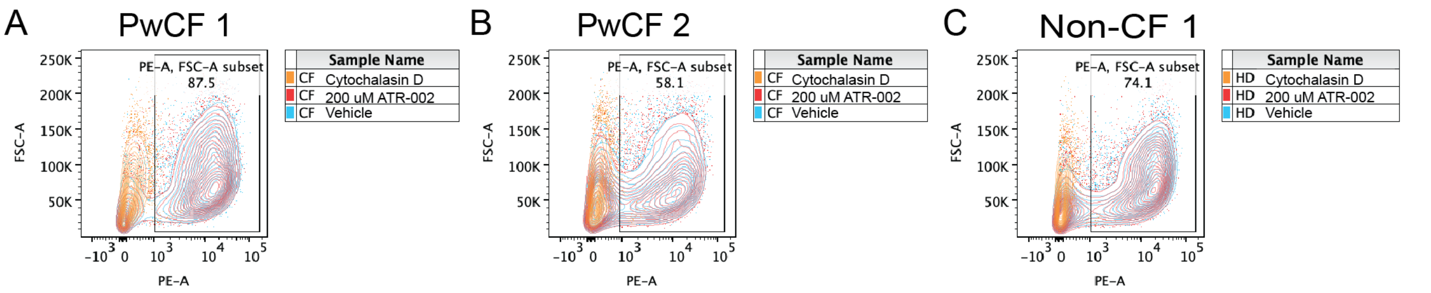

Supplemental Figure 2. Phagocytosis of *S. aureus* by non-CF and CF human monocyte derived macrophages.

Human monocyte-derived macrophages (MDM) from PwCF (A-B) or a non-CF healthy donor (C) were cultured and used in the same phagocytosis experiment of serum opsonized *S. aureus* pHrodo-red bioparticles. Flow cytometry plots are included to demonstrate the overall variability observed between biological samples for the percent of macrophages pHrodo-red-positive (A) 87.5%, (B) 58.1%, or (C) 74.1%. There were no significant differences between vehicle or ATR-002 treated sample for each biological sample. Data from A-B are included in Figure 3D.

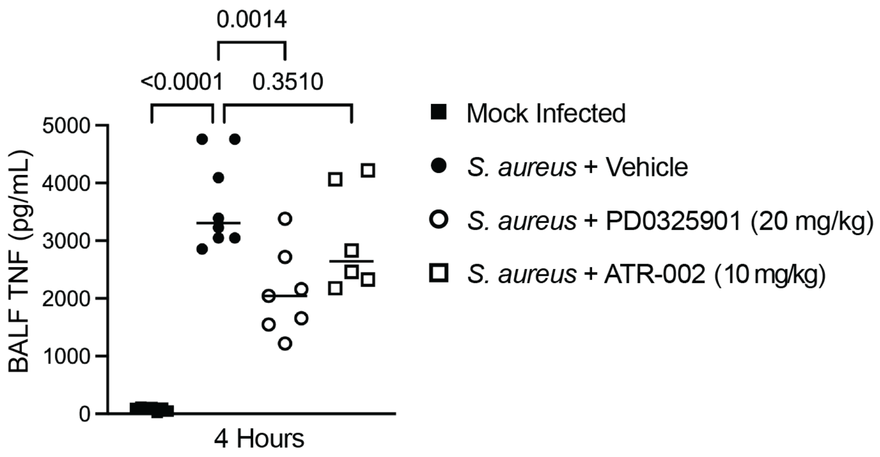

Supplemental Figure 3. Quantification of TNF in BALF 4 Hours after *Staphylococcus aureus* Infection.

Wild-type mice were mock-infected (intranasal delivery of sterile PBS) or i.p. treated with vehicle, 20 mg/kg PD0325901 or 10 mg/kg ATR-002 and infected with 1 x 10^7 CFU of *S. aureus* USA300 by intranasal instillation. Data include male and female animals from n=2 independent experiments and bars represent the mean. Statistical comparisons used one-way ANOVA with Tukey's multiple comparisons tests.

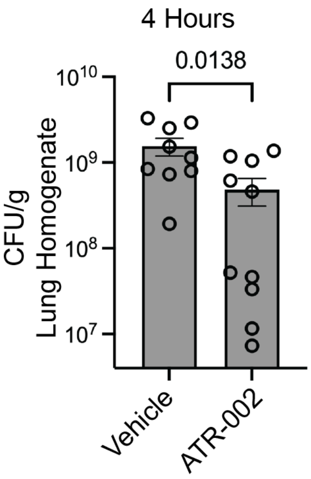

Supplemental Figure 4. Anti-bacterial effects of MEK1/2 inhibitor ATR-002 in *Mek2^{KO}* mice.

Mek2^{KO} mice were provided i.p. treatment with either vehicle or 10 mg/kg ATR-002 and then infected with 1 x 10^7 CFU of *S. aureus* USA300 by intranasal instillation. Bacterial burdens were enumerated from left lung homogenates prepared 4 hours after infection. CFU were normalized for gram of tissue collected. Data include male and female animals from n=2 independent experiments and bars represent the mean $\pm$ SEM. Data in did not have a Gaussian distribution therefore a Kruskal-Wallis with Dunn's multiple comparisons test was used for statistical comparisons.

**Supplemental Table 1. Reagents used in this study.**		
**Item**	**Catalog #**	**Source**
PD0325901	444968	Sigma
CI-1040	PZ0181	Sigma
Trametinib	S2673	Selleckchem
ATR-002	SML3472	Sigma
Human AB Serum	H6914	Sigma
Pam3CSK4	tlrl-pms	InvivoGen
FSL-1	tlrl-fsl	InvivoGen
pHrodo Red *S. aureus*	A10010	ThermoFisher
Cytochalasin-D	C2618	Sigma
Human M-CSF	300-25	Peprotech
p-P44/42 MAPK (ERK1/2) (T202/Y204) antibody	9101	Cell Signaling
P44/42 MAPK (ERK1/2) (137F5)	4695	Cell Signaling
GAPDH (14C10) antibody	2118	Cell Signaling
Anti-Rabbit IgG, HRP-Linked antibody	7074	Cell Signaling
Mueller Hinton Broth 2	90922	Sigma
Brain Heart Infusion Broth	53286	Sigma
Tryptic Soy Agar	22091	Sigma
SCFM1 Media	10001	Synthbiome
Phosphate-Buffered Saline	21-040-CV	Corning
Cell Staining Buffer	420201	Biolegend
Fetal Bovine Serum	F1051	Sigma
RPMI-1640	61870036	ThermoFisher
Human TNF ELISA	DY210-05	Biotechne, R&D Systems
Human CXCL8/IL-8 ELISA	DY208-05	Biotechne, R&D Systems

Mouse TNF ELISA	DY410-05	Biotechne, R&D Systems
Mouse CXCL1 ELISA	DY453-05	Biotechne, R&D Systems
